# Intersection of race/skin color and sex in intentional self-inflicted injury rates among Brazilian people (2014-2023)

**DOI:** 10.1590/0102-311XEN097725

**Published:** 2026-01-01

**Authors:** Gabriel Calegaro, Bruna Gonçalves-Silva, Helen Gonçalves

**Affiliations:** 1 Programa de Pós-graduação em Epidemiologia, Universidade Federal de Pelotas, Pelotas, Brasil.

**Keywords:** Self-Injurious Behavior, Intersectionally, Young Adult, Intention, Comportamento Autodestrutivo, Interseccionalidade, Adulto Jovem, Intenção, Conducta Autodestrutiva, Interseccionalidad, Adulto Joven, Intención

## Abstract

Suicidal and self-injurious behaviors are major public health issues, disproportionately affecting youth. While global suicide rates declined, Brazil has seen a significant rise over recent decades. Considering the persistent social inequality in Brazil, especially among Black/Mixed-race women, an intersectional approach is essential to understanding how the combination of social identities impacts health. This study analyzes reported intentional self-inflicted injury rates among Brazilian people (18-30 years) from 2014 to 2023, according to race/skin color, sex, and their intersection. Data were obtained from official public records (Brazilian Information System for Notifiable Diseases). Intentional self-inflicted injury rates were estimated nationally and by macroregion, for the population aged 18 to 30 years, according to race/skin color, sex, and their intersection. Brazilian national intentional self-inflicted injury rates increased 638% (24.2 to 178.5/100,000), with regional increases up to 969%. Females had higher rates than males, and Mixed-race individuals showed the steepest rise (890%). Intersectional analysis revealed that White females had the highest rates, but Mixed-race females experienced the most significant increase (914%). Regional variations were notable, Mixed-race females led in the North, Northeast and Central-West, while White females dominated the South and Southeast. Intentional self-inflicted injury rates surged among Brazilian people (18-30 years), particularly White/Mixed-race women and Mixed-race individuals, with substantial regional variation. Intersectional and contextual factors (healthcare access, gender, and socioeconomic) are likely contributors. These findings suggest combined effects of racial and gender inequities, policy responses must address intersectional vulnerabilities.

## Abstract (pt)

Comportamentos suicidas e autolesivos são um grande problema de saúde pública que afeta desproporcionalmente os jovens. Enquanto as taxas globais de suicídio diminuíram, o Brasil viu um aumento significativo em décadas passadas. Dada a sua persistente desigualdade social (especialmente entre mulheres pretas/pardas), uma abordagem interseccional é essencial para compreender como a combinação de identidades sociais impacta a saúde. Este estudo analisou as taxas de lesões autoprovocadas intencionalmente relatadas por brasileiros (18-30 anos) entre 2014 e 2023 segundo raça/cor da pele, sexo e sua intersecção. Os dados foram obtidos de registros públicos oficiais (Sistema de Informação de Agravos de Notificação). As taxas de lesões autoprovocadas intencionalmente foram estimadas nacionalmente e por macrorregião para aqueles entre 18 e 30 anos de idade e segundo raça/cor da pele, sexo e sua intersecção. As taxas nacionais de lesões autoinfligidas intencionalmente aumentaram 638% (24,2 para 178,5/100.000), com aumentos regionais de até 969%. As mulheres apresentaram taxas mais altas do que os homens, enquanto os pardos, o aumento mais acentuado (890%). A análise interseccional revelou que as mulheres brancas apresentaram as taxas mais altas e as mulheres pardas, o aumento mais significativo (914%). As variações regionais foram notáveis; as pardas mostraram taxas mais altas no Norte, Nordeste e Centro-oeste enquanto as brancas, no Sul e o Sudeste. As taxas de lesões autoprovocadas intencionalmente aumentaram entre os brasileiros (18-30 anos), particularmente entre brancas/pardas e pardos, com variação regional substancial. Fatores interseccionais e contextuais (acesso à saúde, gênero e socioeconômicos) provavelmente contribuem a esses números. Essas descobertas sugerem efeitos combinados de desigualdades raciais e de gênero. Políticas devem abordar vulnerabilidades interseccionais.

## Abstract (es)

Las conductas suicidas y autolesivas constituyen un grave problema de salud pública que afecta de manera desproporcionada a los jóvenes. Si bien las tasas mundiales de suicidio han disminuido, en Brasil se ha registrado un aumento significativo en las últimas décadas. Teniendo en cuenta la persistente desigualdad social en Brasil,especialmente entre las mujeres negras y morenas,es esencial adoptar un enfoque interseccional para comprender cómo la combinación de identidades sociales repercute en la salud. Este estudio analiza las tasas de autolesiones intencionales notificadas entre la población brasileña (18-30 años) entre 2014 y 2023, según la raza/color de piel, el sexo y su intersección. Los datos se obtuvieron de registros públicos oficiales (Sistema de Información sobre Enfermedades Notificables). Las tasas de autolesiones intencionales se estimaron a nivel nacional y por macrorregión, para la población de personas de entre 18 y 30 años y según la raza/color de piel, el sexo y su intersección. Las tasas nacionales de lesiones autoinfligidas intencionales aumentaron un 638% (de 24,2 a 178,5/100.000), con aumentos regionales de hasta el 969%. Las mujeres presentaron tasas más altas que los hombres, y las personas de raza morena mostraron el aumento más pronunciado (890%). El análisis interseccional reveló que las mujeres blancas tenían las tasas más altas,pero las mujeres morenas experimentaron el aumento más significativo (914%). Las variaciones regionales fueron notables: las mujeres de raza negra lideraron en el Norte, Noreste y Centro-oeste, mientras que las mujeres de raza blanca dominaron en el Sur y Sureste. Las tasas de lesiones autoinfligidas intencionales aumentaron entre la población brasileña (18-30 años), en particular entre las mujeres de raza blanca/negra y las personas morenas, con variaciones regionales sustanciales. Es probable que los factores interseccionales y contextuales (acceso a la atención sanitaria, género y socioeconómicos) contribuyan a ello. Estos hallazgos sugieren efectos combinados de las desigualdades raciales y de género, por lo que las respuestas políticas deben abordar las vulnerabilidades interseccionales.

## Introduction

Globally, suicide has become a major public health issue. It is estimated that around 700,000 people died by suicide in 2021, and it is one of the four main causes of death among young people (15-29 years), reflecting its disproportionate impact on youth ^1^ ^,^ ^2^ ^,^ ^3^. Moreover, for every death by suicide, it is estimated that at least 20 other people have attempted suicide at least once in their lifetime ^2^ ^,^ ^3^. Both suicide and suicide attempts are preventable, and due to its recognition as a significant public health issue by the World Health Organization (WHO), the United Nations (UN) included a one-third reduction in suicide mortality as one of the objectives of the Sustainable Development Goals (target 3.4) ^1^ ^,^ ^3^.

Despite a 36% global decline in suicide rates from 2000 to 2019, the American continent had an annual increase of 1.25% during the same period ^4^ ^,^ ^5^. In 2019, over 97,000 people died by suicide in the region, with pronounced gender disparities. The age-standardized rates were 9.0/100,000, being higher in males (14.2/100,000) than females (4.1/100,000) ^5^. Brazil reflects this regional pattern, with a 70% increase in suicide deaths over the last decades (1990s-2010s), resulting in a 49% increase in the suicide rate (from 4.3 deaths by suicide per 100,000 inhabitants to 6.4/100,000) ^6^. Suicide now represents the second leading cause of death among Brazilian adolescents (15-19 years) and the fourth among young adults (20-29 years) ^7^.

For suicide attempts, screening in Brazil is conducted via reports of intentional self-inflicted injury by the Surveillance System for Violence and Accidents (VIVA, acronym in Portuguese) of the Brazilian Information System for Notifiable Diseases (SINAN, acronym in Portuguese). In Brazil, reporting any case treated by the public health system has been mandatory since 2009, and those treated by the private health system since 2019. Although underreported, there has been an increase in reported cases over the last decade (497.5%) ^8^. According to the latest report from the Brazilian Ministry of Health (2024) ^9^, 52% of the reported cases in 2021 occurred among people aged 15 to 29 years, 70% among women, and 45% among Black/Mixed-race individuals. In addition, a recent study on intentional self-inflicted injury notifications in Brazil over the last decade (2011-2022), using SINAN data, showed that individuals aged 10 to 59 years had the highest increase in self-harm notification rates ^10^.

The risk of suicide attempts is multifactorial, influenced by a range of various factors, including biological and psychological ^11^ ^,^ ^12^. Biological factors (such as methylation of genes associated with stress, changes in neuroplasticity and dysregulation of neurotransmitters), along with psychological factors (such as anxious, depressive, aggressive, or impulsive behaviors and psychological stress), can contribute to the development of suicidal and self-injurious behaviors ^11^ ^,^ ^12^. Previous engagement in self-injurious behaviors, such as intentional self-inflicted injury, is associated with a higher risk of suicide attempts ^11^ ^,^ ^12^ ^,^ ^13^.

In the sociodemographic dimension, being female and/or a racial minority is associated with a higher risk of suicide attempts, a pattern attributed to lack of access to health promotion resources, social instability, and adverse events (e.g., discrimination experienced throughout life due to sexism and racism, which remain prevalent in many places) ^11^ ^,^ ^12^ ^,^ ^14^. Moreover, lower family income and educational levels are also associated with higher suicide attempt risk ^11^ ^,^ ^12^. 

In the Brazilian sociohistorical context, deeply rooted in slavery and colonialism, racism and sexism have shaped societal structures since the nation’s foundation ^15^ ^,^ ^16^ ^,^ ^17^. As the last country in the Americas to abolish slavery (1888), Brazil received approximately 40% of all Africans trafficked during the transatlantic slave trade ^18^, leaving a legacy in which descendants of enslaved Africans − comprising 55% of the population (Black = *pretos* and Mixed-race = *pardos*, collectively termed “população negra”) − remain a racial minority, contrasted with the White majority (43%) ^19^. While debates persist over discrepancies between official racial categorizations and self-identification, particularly regarding pardos, who often resist binary labels yet face discrimination akin to Black Brazilians ^20^, this study acknowledges but does not delve into classification complexities, focusing instead on systemic inequities perpetuated by Brazil’s historical and racial dynamics.

Despite significant social advances, Black/Mixed-race women still face lower income, reduced educational attainment, and higher unemployment rates compared to White men, reinforcing structural vulnerabilities ^17^ ^,^ ^21^ ^,^ ^22^ ^,^ ^23^ ^,^ ^24^.

Considering the burden of suicidal and self-injurious outcomes among youth in Brazil and the local sociohistorical context, it is important to investigate differences in intentional self-inflicted injury rates according to race/skin color and sex, and to explore differences based on their intersection. Even though current knowledge shows a higher risk of suicide attempts among females and racial minority populations, little is known about the cumulative effects of multiple social identities on suicide attempts, given that most studies have analyzed these social identities separately ^25^ ^,^ ^26^. 

Intersectionality theory provides a lens to explore the dynamics of multiple social identities and macrosocial systems of power and oppression on health outcomes ^27^ ^,^ ^28^. As proposed by this theory, the combination of social identities cannot be experienced separately or at the individual level, as this would not reflect the influence of macrosocial systems of power and oppression, such as racism and sexism, nor their amplified effects on health outcomes such as suicidal outcomes ^26^ ^,^ ^27^ ^,^ ^28^. For instance, being a Black/Mixed-race woman in Brazil may entail synergistic exposure to racial and gender discrimination, potentially intensifying the risk of intentional self-inflicted injury and suicidal outcomes.

In addition, most evidence on suicidal and intentional self-injurious outcomes comes from high-income countries (HICs); however, around 80% of all suicides occur in low- and middle-income countries (LMICs), such as Brazil, where knowledge is still scarce ^29^. Given this knowledge gap, the availability of a public database on intentional self-inflicted injury in Brazil and the higher occurrence of self-harm notifications among Brazilian youth and adults over the last decade, we aim to assess the rates of intentional self-inflicted injury among Brazilian people aged 18 to 30 years according to race/skin color, sex, and their intersection over the last decade (2014-2023).

## Methods

### Study design

This is a descriptive cross-sectional study of intentional self-inflicted injuries recorded among Brazilians aged 18 to 30 years from 2014 to 2023. Although the standard classification of young adults ranges from 18 to 29 years, evidence suggests that important stages of neurological development are consolidated in the late 20s and early 30s ^30^; thus, it was opted to include only those aged 18 to 30 years. 

Data on intentional self-inflicted injuries were obtained from the SINAN, a digital database of the Brazilian Unified National Health System (SUS, acronym in Portuguese). Data collection protocols were standardized in 2014; thus, this analysis covers 2014-2023 to maintain comparability. All cases of intentional self-inflicted injury treated by healthcare professionals (in any public or private facility across the country) are mandatorily reported to SINAN using the *Interpersonal/Self-Inflicted Violence* form, and must be categorized following the International Classification of Diseases, 10th revision (ICD-10), codes X60 to X847,9. The form is available at: http://portalsinan.saude.gov.br/images/documentos/Agravos/via/violencia_v5.pdf.

Through this registry, the following variables were extracted: age group, educational attainment, macroregion of occurrence, race/skin color, sex, and intentional self-inflicted injury. Data on the place of occurrence, type of injury (suicidal or non-suicidal), method used, and motivation are also available in the SINAN but were not used in this study. Although it is possible to distinguish between attempted suicide and non-suicidal intentional self-injury based on the motivation recorded in each case (i.e., whether the objective was to take one’s own life or simply to cause harm without lethal intent), this specific classification was not performed due to the high number of incomplete records or insufficient information in the report forms. Therefore, to ensure greater accuracy and comparability of data, all cases of intentional self-inflicted injury were considered, following the criteria adopted by the Brazilian Ministry of Health.

Population estimates were obtained from the national Census of 2010 and 2022, conducted by the Brazilian Institute of Geography and Statistics (IBGE, acronym in Portuguese). 

### Measures

#### Exposures

Race/skin color and sex were used as exposures. The race/skin color recorded in the form is based on the patient’s self-report, meaning it is self-identified by the individual who attempted suicide. The response options follow the racial classification of the national Census (White, Black, Mixed-race, Yellow, and Indigenous). Sex (male or female) was also inquired about by the healthcare professional directly from the patient. Although the SINAN form includes an item for gender identity, biological sex was preferred due to the low rate of missing information for biological sex compared to the high rate of missing or poorly completed information for gender identity. 

For this study, only data regarding intentional self-inflicted injury among White, Black, and Mixed-race individuals will be analyzed when stratifying by race/skin color. Even though the Brazilian indigenous population is also a racial minority group with several social and political disadvantages, the dynamics of racial relations between Indigenous people and White people have a different sociohistorical context compared to the Afro-Brazilian population ^31^. Additionally, suicide is related to specific social and cultural factors in the Indigenous populations, which affect the epidemiology and etiology of suicidal outcomes ^32^. For these reasons, this matter should be the subject of another study. Regarding the Yellow population (composed primarily of those of East and Southeast Asian ancestry), the structural racism of Brazilian society does not affect them to the same extent as the Black/Mixed-race and Indigenous populations ^33^.

In this analysis, considering the limitations of the dataset, we opted to utilize a categorized intersectional approach ^34^, based on a cross-categorical classification of race/skin color and sex. A variable was created by combining the categories of these variables, resulting in six categories: White male, Black male, Mixed-race male, White female, Black female, and Mixed-race female. The group with the most social privilege (White male) was used as the reference group. Individuals who did not have complete data for at least one of the two variables were excluded from the intersectional cross-categorization variable.

The macroregion of occurrence of the intentional self-inflicted injury was used as a stratification variable. Brazilian territory is divided into five macroregions: North, Northeast, Southeast, South, and Central-West.

#### Outcome

For this study, the outcome comprised all cases reported as intentional self-inflicted injury, using the ICD-10 criteria (X60-X84) adopted by the Brazilian Ministry of Health. The nature and type of the patient’s injury are assessed by a healthcare professional during the patient’s visit to any public or private healthcare facility. To complete the SINAN report form, the healthcare professional must specify two items: the type of injury (item 2; ICD-10) and whether it was self-inflicted (Item 54: Was the injury self-inflicted? (1) Yes; (2) No; (9) Unknown).

The rate of intentional self-inflicted injury was estimated for the general population aged 18 to 30 years and for all exposure variables. For population estimates, data from the IBGE’s population projection were obtained for the general population aged 18 to 30 years and its distribution by sex for each year of the study period ^35^. The population estimates for race/skin color were estimated by multiplying the general population estimate for each year by the percentage of each race/skin color category according to the national census data. The population estimates for the intersection of race/skin color and sex were calculated by multiplying the general population estimate for each year by the percentage of each intersectional category according to the Brazilian census data. From 2014 to 2019, data from the 2010 Census were used for these estimates, and from 2020 to 2023, data from the 2022 Census (available at: https://sidra.ibge.gov.br/home/cnt/brasil).

### Statistical analysis

Initial descriptive analyses characterized intentional self-inflicted injury reports according to key sociodemographic exposures (educational attainment, race/skin color, and sex), with particular attention to data completeness in each variable. Due to a high proportion of missing data for educational attainment (24-32% nationally, see Supplementary Material: Table S1; https://cadernos.ensp.fiocruz.br/static//arquivo/suppl-e00097725_7755.pdf), this variable was excluded from the main analysis to maintain analytical rigor and minimize potential bias introduced by incomplete records.

Intentional self-inflicted injury rates were estimated for all Brazilian territory and its macroregions. Furthermore, intentional self-inflicted injury rates were estimated for race/skin color, sex, and their intersection. All rates were calculated for each year of the study period, and all analyses were stratified by the macroregion where the intentional self-inflicted injury occurred. The increase in rates over the ten years was estimated as the percentual difference between the 2023 and the 2014 rates.

A trend test (Kruskal-Wallis test) was conducted to assess temporal trends in intentional self-inflicted injury for all Brazilian territory, macroregions, and according to race/skin color, sex, and their intersection during the study period. Brazilian national and regional differences in intentional self-inflicted injury rates were assessed with a chi-square test for each year of the study period.

All rates were estimated using Microsoft Excel, version 2021 (https://products.office.com/), and all statistical tests were performed using Stata, version 15.0 (https://www.stata.com). Statistical significance was set at 5%. For each variable, analyses were conducted considering only cases with complete data; all cases with missing data were excluded.

### Ethical considerations

Since this study was conducted utilizing secondary data obtained from public records, approval by a research ethics committee was not required. All data were derived from public databases obtained via the websites of the Brazilian Ministry of Health and IBGE.

## Results

Table 1 presents the rates of reported intentional self-inflicted injury among Brazilian people (18-30 years) from 2014 to 2023 for all of Brazil and for each macroregion. In 2014, the national rate of intentional self-inflicted injury was 24.2/100,000, and by 2023, the rate had increased by 638%, reaching 178.5/100,000. During 2014-2023, all regions showed an increase in their rates: 969% − Central-West; 928% − Northeast; 625% − North; 605% − Southeast; and 451% − South (Table 1). From 2014 to 2023, the highest rates were observed in the Southeast, South, and Central-West regions. By 2023, the Central-West Region showed the highest rate. The lowest rates over the ten years were found in the North Region.

**Table 1:** Reported intentional self-inflicted injury rate (per 100,000) among Brazilian adults (18-30 years) nationwide and by regions from 2014 to 2023.

Year	Brazil *	North *		Northeast *		Southeast *		South *		Central-West *	
		Rate **	p-value	Rate **	p-value	Rate **	p-value	Rate **	p-value	Rate **	p-value
2014	24.2	11.6	< 0.001	11.3	< 0.001	30.5	< 0.001	40.6	< 0.001	23.3	0.292
2015	31.8	16.8	< 0.001	15.5	< 0.001	39.1	< 0.001	54.2	< 0.001	30.1	0.088
2016	55.1	18.6	< 0.001	16.9	< 0.001	45.1	< 0.001	61.7	< 0.001	34.8	0.176
2017	76.1	26.7	< 0.001	27.1	< 0.001	66.8	< 0.001	101.3	< 0.001	46.3	< 0.001
2018	76.4	32.3	< 0.001	40.2	< 0.001	89.9	< 0.001	139.4	< 0.001	76.7	0.848
2019	113.8	54.3	< 0.001	67.5	< 0.001	124.5	< 0.001	199.0	< 0.001	143.6	< 0.001
2020	92.1	40.9	< 0.001	52.4	< 0.001	103.9	< 0.001	150.8	< 0.001	129.9	< 0.001
2021	110.3	51.8	< 0.001	71.8	< 0.001	127.9	< 0.001	151.8	< 0.001	153.0	< 0.001
2022	130.9	63.4	< 0.001	83.5	< 0.001	157.2	< 0.001	168.6	< 0.001	179.7	< 0.001
2023	178.5	84.1	< 0.001	116.2	< 0.001	215.0	< 0.001	223.7	< 0.001	249.1	< 0.001
Difference (%)	638	625		928		605		451		969	

* p < 0.001 (trend);

** Rate per 100,000 inhabitants.

Regarding sex, throughout the period, females exhibited the highest rates of intentional self-inflicted injury in the country, and the rate increase was higher for females (672%) than for men (587%) (Table 2). When examining the data by region (Table 3), except for the North, this trend remained consistent among females.

**Table 2:** Reported intentional self-inflicted injury rate (per 100,000) among Brazilian adults (18-30 years) by sex and race/skin color from 2014 to 2023.

Year	Male *	Female *	p-value	White *	Black *	Mixed-race *	p-value
2014	16.9	31.3	< 0.001	25.5	17.7	16.5	< 0.001
2015	23.1	40.3	< 0.001	31.8	23.4	22.6	< 0.001
2016	25.7	46.7	< 0.001	37.2	24.7	26.5	< 0.001
2017	37.8	72.3	< 0.001	58.3	38.8	42.3	< 0.001
2018	52.1	100.6	< 0.001	80.5	50.8	60.5	< 0.001
2019	72.4	155.2	< 0.001	114.9	76.9	94.8	< 0.001
2020	61.6	122.6	< 0.001	103.7	54.6	72.4	< 0.001
2021	71.4	149.3	< 0.001	117.0	64.6	92.5	< 0.001
2022	85.0	177.0	< 0.001	137.0	79.1	66.2	< 0.001
2023	116.1	241.5	< 0.001	187.7	119.3	163.3	< 0.001
Difference (%)	587	672		636	574	890	

* p < 0.001 (trend).

**Table 3:** Reported intentional self-inflicted injury rate (per 100,000) among Brazilian adults (18-30 years) by sex and race/skin color from 2014 to 2023, stratified by region.

Region/Year	Male *	Female *	p-value	White *	Black *	Mixed-race *	p-value
North							
2014	7.8	15.3	< 0.001	7.1	6.9	11.5	< 0.001
2015	13.2	20.4	< 0.001	6.6	8.2	18.5	< 0.001
2016	14.8	22.4	< 0.001	9.5	11.6	20.3	< 0.001
2017	18.6	34.8	< 0.001	14.9	21.8	27.8	< 0.001
2018	20.9	43.8	< 0.001	18.8	20.7	34.6	< 0.001
2019	36.9	71.8	< 0.001	37.4	32.7	56.5	< 0.001
2020	28.5	53.4	< 0.001	23.7	20.9	43.4	< 0.001
2021	35.1	68.6	< 0.001	31.1	23.5	57.3	< 0.001
2022	44.3	82.8	< 0.001	34.3	29.7	71.2	< 0.001
2023	60.0	108.5	< 0.001	50.9	43.3	93.1	< 0.001
Difference (%)	669	609		617	528	710	
Northeast							
2014	7.4	15.0	< 0.001	5.6	6.0	9.7	< 0.001
2015	10.1	20.6	< 0.001	6.2	7.8	14.6	< 0.001
2016	12.3	21.4	< 0.001	6.9	8.2	16.4	< 0.001
2017	19.2	34.7	< 0.001	13.2	11.4	28.5	< 0.001
2018	27.0	53.1	<0.001	20.9	17.1	44.8	< 0.001
2019	43.3	91.2	< 0.001	41.4	34.1	72.0	< 0.001
2020	35.4	69.1	< 0.001	40.1	21.3	57.1	< 0.001
2021	44.9	98.5	< 0.001	53.2	30.2	77.9	< 0.001
2022	53.5	113.3	< 0.001	61.1	36.2	89.8	< 0.001
2023	72.4	159.8	< 0.001	86.1	53.2	127.4	< 0.001
Difference (%)	878	965		1,438	787	1,213	
Southeast							
2014	21.2	39.8	< 0.001	26.9	27.7	23.9	< 0.001
2015	28.4	49.8	< 0.001	33.3	34.9	30.8	0.004
2016	31.0	59.2	< 0.001	39.7	36.1	36.9	0.004
2017	46.0	87.6	< 0.001	58.9	56.1	60.4	0.102
2018	59.8	120.0	< 0.001	80.9	72.0	81.3	< 0.001
2019	76.7	172.4	< 0.001	109.0	102.2	119.2	< 0.001
2020	67.3	140.5	< 0.001	105.0	74.0	88.3	< 0.001
2021	80.6	175.7	< 0.001	128.9	87.6	110.6	< 0.001
2022	98.0	217.0	< 0.001	156.1	113.8	147.4	< 0.001
2023	135.3	295.6	< 0.001	217.7	171.9	204.8	< 0.001
Difference (%)	538	643		709	521	757	
South							
2014	29.3	52.0	< 0.001	43.2	32.3	23.0	< 0.001
2015	40.8	67.7	< 0.001	55.4	50.1	36.5	< 0.001
2016	45.0	78.6	< 0.001	63.2	55.8	41.5	< 0.001
2017	68.3	134.7	< 0.001	103.0	96.9	66.0	< 0.001
2018	98.9	180.3	< 0.001	142.1	128.9	96.4	< 0.001
2019	131.9	266.9	< 0.001	200.5	181.9	147.6	< 0.001
2020	106.2	196.0	< 0.001	174.1	121.0	83.8	< 0.001
2021	105.2	199.3	< 0.001	171.7	130.0	84.2	< 0.001
2022	120.5	217.5	< 0.001	192.6	123.0	89.7	< 0.001
2023	158.8	289.9	< 0.001	252.0	195.5	129.8	< 0.001
Difference (%)	442	458		483	505	464	
Central-West							
2014	16.8	29.7	< 0.001	18.2	11.8	20.9	0.004
2015	22.1	38.0	< 0.001	21.7	20.6	23.4	0.468
2016	23.3	46.3	< 0.001	24.1	19.4	30.6	< 0.001
2017	29.4	63.4	< 0.001	40.9	29.4	41.5	0.014
2018	54.5	99.1	< 0.001	54.4	43.9	67.6	< 0.001
2019	88.7	199.1	< 0.001	94.1	79.1	128.3	< 0.001
2020	83.8	176.6	< 0.001	88.8	61.7	104.3	< 0.001
2021	101.2	205.5	< 0.001	100.3	65.0	138.3	< 0.001
2022	115.6	244.7	< 0.001	117.5	80.7	174.5	< 0.001
2023	163.9	336.1	< 0.001	172.7	120.1	272.9	< 0.001
Difference (%)	876	1,032		849	918	1,206	

* p < 0.001 (trend).

Regarding race/skin color, the White population maintained the highest rate until 2015; however, from 2016 to 2023, the rate for the Mixed-race population rose to the second highest, followed by the Black population (Table 2). In addition, from 2014 to 2023, rates increased for all groups: Mixed-race (890%), White (636%), and Black (574%). This pattern varied by region (Table 3). 

The White population had the highest rates in the Southeast and South regions and the second-highest rate increase. The North and Northeast regions experienced the highest rate increase among White individuals, whereas the Central-West Region showed the lowest increase. The Black population had the lowest rates and the smallest rate increase across all regions, except for the South, where they had the second highest rate and the largest increase. The Mixed-race population had the highest rates in the North, Northeast, and Central-West regions and the second highest in the Southeast. They also had the highest rate increase in the Southeast and Central-West, the second highest in the North and Northeast, and the lowest in the South.

Table 4 presents the rates of reported intentional self-inflicted injury according to the intersection of race/skin color and sex. From 2014 to 2023, White and Mixed-race females had the highest rates, followed by Black females. In contrast, male subgroups − particularly Mixed-race and Black males − had the lowest rates. The rate differences for Black and Mixed-race males and for all three female groups (White, Black, and Mixed-race), compared with White males, were significant over the ten years (2014-2023). Moreover, the highest rate increase was observed among Mixed-race females (914%), followed by Mixed-race males (839%), White females (672%), White males (565%), Black males (518%), and Black females (494%). Results varied by region (Table 5).

**Table 4:** Reported intentional self-inflicted injury rate (per 100,000) among Brazilian adults (18-30 years) by the intersection of race/skin color and sex from 2014 to 2023.

Year	White male*	Black male*		Mixed-race male*		White female*		Black female*		Mixed-race female*	
	Reference	Rate **	p-value	Rate **	p-value	Rate **	p-value	Rate **	p-value	Rate **	p-value
2014	18.2	13.0	< 0.001	11.5	< 0.001	32.5	< 0.001	27.3	< 0.001	21.6	< 0.001
2015	23.8	18.0	< 0.001	16.4	< 0.001	39.4	< 0.001	35.1	< 0.001	28.9	< 0.001
2016	26.2	20.2	< 0.001	19.2	< 0.001	47.6	< 0.001	35.6	< 0.001	34.0	< 0.001
2017	39.6	30.5	< 0.001	29.9	< 0.001	76.0	< 0.001	57.3	< 0.001	55.1	< 0.001
2018	54.3	40.0	< 0.001	41.7	< 0.001	105.2	< 0.001	75.1	< 0.001	79.8	< 0.001
2019	72.9	55.1	< 0.001	60.4	< 0.001	154.7	< 0.001	119.8	< 0.001	130.0	< 0.001
2020	68.3	37.7	< 0.001	48.6	< 0.001	137.1	< 0.001	73.2	< 0.001	96.4	< 0.001
2021	74.2	43.4	< 0.001	61.2	< 0.001	157.6	< 0.001	88.0	< 0.001	124.1	< 0.001
2022	87.4	53.1	< 0.001	75.9	< 0.001	184.0	< 0.001	107.7	< 0.001	154.6	< 0.001
2023	121.0	80.3	< 0.001	108.0	< 0.001	250.8	< 0.001	162.2	< 0.001	219.0	< 0.001
Difference (%)	565	518		839		672		494		914	

* p < 0.001 (trend);

** Rate per 100,000 inhabitants.

**Table 5:** Reported intentional self-inflicted injury rate (per 100,000) among Brazilian adults (18-30 years) by the intersection of race/skin color and sex from 2014 to 2023, stratified by region.

Region/Year	White male *	Black male *		Mixed-race male *		White female *		Black female *		Mixed-race female *	
	Reference	Rate **	p-value	Rate **	p-value	Rate **	p-value	Rate **	p-value	Rate **	p-value
North											
2014	3.5	5.0	0.423	6.9	0.012	10.5	< 0.001	9.6	0.007	16.1	< 0.001
2015	3.5	3.7	0.904	14.3	< 0.001	9.4	< 0.001	14.3	< 0.001	22.7	< 0.001
2016	6.1	7.4	0.579	16.2	< 0.001	12.7	< 0.001	17.4	< 0.001	24.5	< 0.001
2017	8.8	15.5	0.024	19.0	< 0.001	20.7	< 0.001	30.7	< 0.001	36.7	< 0.001
2018	8.5	12.3	0.175	21.4	< 0.001	28.5	< 0.001	32.2	< 0.001	47.9	< 0.001
2019	21.7	23.3	0.710	38.8	< 0.001	52.1	< 0.001	46.2	< 0.001	74.5	< 0.001
2020	14.8	16.5	0.600	29.4	< 0.001	32.3	< 0.001	26.2	< 0.001	57.2	< 0.001
2021	15.5	17.4	0.571	39.5	< 0.001	46.0	< 0.001	30.8	< 0.001	74.9	< 0.001
2022	15.2	19.3	0.240	52.1	< 0.001	52.6	< 0.001	42.4	< 0.001	89.8	< 0.001
2023	32.9	32.9	0.998	66.5	< 0.001	68.2	< 0.001	55.8	< 0.001	119.1	< 0.001
Difference (%)	840	558		864		549		481		640	
Northeast											
2014	3.0	5.0	0.026	6.6	< 0.001	7.9	< 0.001	9.1	< 0.001	12.8	< 0.001
2015	4.0	6.7	0.006	9.5	< 0.001	8.2	< 0.001	11.5	< 0.001	19.7	< 0.001
2016	4.2	7.1	0.005	12.4	< 0.001	9.4	< 0.001	12.1	< 0.001	20.4	< 0.001
2017	8.6	9.1	0.726	21.1	< 0.001	17.4	< 0.001	17.7	< 0.001	35.8	< 0.001
2018	11.9	14.0	0.200	31.2	< 0.001	29.3	< 0.001	25.9	< 0.001	58.3	< 0.001
2019	24.2	27.8	0.118	46.9	< 0.001	57.3	< 0.001	52.1	< 0.001	96.8	< 0.001
2020	18.7	16.9	0.342	38.6	< 0.001	43.9	< 0.001	25.8	< 0.001	74.8	< 0.001
2021	22.6	19.1	0.073	49.1	< 0.001	60.3	< 0.001	42.0	< 0.001	105.4	< 0.001
2022	26.2	26.3	0.950	58.1	< 0.001	69.1	< 0.001	46.7	< 0.001	120.1	< 0.001
2023	38.0	36.6	0.613	80.9	< 0.001	96.3	< 0.001	70.8	< 0.001	169.3	< 0.001
Difference (%)	1,167	632		1,126		1,119		678		1,223.0	
Southeast											
2014	19.0	20.7	0.290	17.2	0.068	34.4	< 0.001	43.7	< 0.001	30.9	< 0.001
2015	25.4	26.4	0.606	22.3	< 0.001	40.7	< 0.001	54.8	< 0.001	39.7	< 0.001
2016	27.5	30.7	0.112	24.9	0.028	51.4	< 0.001	52.8	< 0.001	49.5	< 0.001
2017	41.2	44.7	0.168	41.5	0.872	75.7	< 0.001	85.3	< 0.001	80.4	< 0.001
2018	53.0	58.1	0.070	54.7	0.305	107.3	< 0.001	108.9	< 0.001	109.4	< 0.001
2019	67.7	70.4	0.410	72.2	0.018	148.1	< 0.001	168.2	< 0.001	168.5	< 0.001
2020	67.5	48.6	< 0.001	57.5	< 0.001	140.1	< 0.001	101.8	< 0.001	120.3	< 0.001
2021	79.5	59.6	< 0.001	71.1	< 0.001	175.4	< 0.001	118.5	< 0.001	151.7	< 0.001
2022	94.8	76.1	< 0.001	93.6	0.597	213.9	< 0.001	155.2	< 0.001	203.3	< 0.001
2023	134.7	111.4	< 0.001	132.5	0.434	295.8	< 0.001	238.1	< 0.001	280.0	< 0.001
Difference (%)	609	438		670		760		445		806	
South											
2014	31.7	20.2	0.017	16.3	< 0.001	54.4	< 0.001	50.3	< 0.001	30.6	0.682
2015	41.1	45.4	0.432	29.9	< 0.001	69.4	< 0.001	62.2	< 0.001	44.1	0.346
2016	46.0	44.8	0.841	31.4	< 0.001	80.1	< 0.001	75.7	< 0.001	52.8	0.045
2017	68.9	78.7	0.172	46.8	< 0.001	136.4	< 0.001	130.6	< 0.001	87.4	< 0.001
2018	101.0	96.7	0.625	72.1	< 0.001	182.4	< 0.001	182.8	< 0.001	123.6	< 0.001
2019	131.8	133.2	0.887	105.1	< 0.001	267.9	< 0.001	261.7	< 0.001	194.9	< 0.001
2020	121.6	86.7	< 0.001	63.4	< 0.001	224.7	< 0.001	160.0	< 0.001	106.5	0.001
2021	118.0	86.5	< 0.001	64.5	< 0.001	223.5	< 0.001	179.5	< 0.001	106.2	0.011
2022	139.5	80.2	< 0.001	65.9	< 0.001	243.6	< 0.001	177.7	< 0.001	116.2	< 0.001
2023	178.7	146.4	0.001	98.6	< 0.001	322.3	< 0.001	251.1	< 0.001	164.3	0.012
Difference (%)	464	625		505		492		399		437	
Central-West											
2014	12.6	8.4	0.198	14.1	0.420	23.4	< 0.001	16.4	0.296	27.8	< 0.001
2015	15.5	12.6	0.413	17.5	0.347	27.5	< 0.001	31.1	< 0.001	29.5	< 0.001
2016	15.4	11.9	0.326	22.7	0.001	32.3	< 0.001	29.4	0.001	38.7	< 0.001
2017	23.0	19.7	0.446	29.3	0.017	57.9	< 0.001	42.3	< 0.001	54.0	< 0.001
2018	35.6	35.2	0.930	48.8	< 0.001	72.2	< 0.001	56.3	0.001	86.8	< 0.001
2019	57.0	57.0	0.992	77.2	< 0.001	129.2	< 0.001	109.1	< 0.001	180.5	< 0.001
2020	50.5	41.6	0.120	67.0	< 0.001	125.0	< 0.001	85.9	< 0.001	141.9	< 0.001
2021	58.5	43.6	0.014	96.7	< 0.001	139.8	< 0.001	90.8	< 0.001	180.2	< 0.001
2022	64.3	51.2	0.044	116.5	< 0.001	168.0	< 0.001	116.3	< 0.001	232.6	< 0.001
2023	105.9	83.5	0.007	181.9	< 0.001	236.0	< 0.001	164.4	< 0.001	364.3	< 0.001
Difference (%)	740	894		1,190		908		902		1,210	

* p < 0.001 (trend);

** Rate per 100,000 inhabitants.

In the North, Northeast, and Central-West, Mixed-race females had the highest rates at both the start and the end of the period (2014 and 2023). They also had the highest rate increase in the Northeast and Central-West, while Mixed-race males had the highest increase in the North. In the Southeast, a reversal occurred: Black females had the highest rate in 2014, but by 2023 they were surpassed by White females. The highest rate increase was among Mixed-race females (806%). In the South, from 2014 to 2023, White females consistently had the highest rates, and Black males exhibited the highest rate increase (625%).

In the North, significant differences were observed for all groups except Black males when compared with White males. In the Northeast, South, and Central-West, rate differences were significant for Mixed-race males and all female groups throughout the whole period or most of it. In the Southeast, significant differences persisted between White males and all female groups across the entire period.

Across all analyses, a positive trend in the rates was observed, with the highest rates in 2023. However, when considering the pre-pandemic period (2014-2019), a peak was observed in 2019, followed by a decrease in 2020 and an increase from 2021 onward. These trends were observed nationwide, across all regions, for males, females, all racial groups, and all intersectional groups.

Dataset characteristics are available in the Supplementary Material (Table S1; https://cadernos.ensp.fiocruz.br/static//arquivo/suppl-e00097725_7755.pdf).

## Discussion

From 2014 to 2023, Brazil experienced a sharp escalation in reported intentional self-inflicted injuries among people aged 18-30 years, with striking regional and demographic disparities. Mixed-race individuals and females − especially Mixed-race females − showed the highest rates and rate increases. These findings reinforce patterns seen globally, in which women tend to engage in self-harm behaviors more frequently than men, though suicide rates are higher among men. Mixed-race individuals had the highest rates in three regions (North, Northeast, and Central-West), while White individuals predominated in the South and Southeast.

Mandatory reporting of intentional self-inflicted injuries (whether suicidal or not), initially restricted to the public health system (2009), was extended to the private sector in 2019 ^36^. This expansion helps explain, in part, the abrupt increase in notifications, and the rise in rates from 2019 onwards, as well as the peak observed in that year. The continuous increase from 2021 onward may have been influenced by the effects of the pandemic, such as social isolation and economic crisis, which disproportionately impacted the most vulnerable groups ^22^. Moreover, the decrease in rates in 2020 can be attributed to social distancing measures during the height of the COVID-19 pandemic. Given that reporting requires seeking care in healthcare services, many cases may have been unreported, as individuals might have avoided healthcare services due to social distancing measures, and a large portion of Brazil's healthcare services were fully dedicated to treating COVID-19 cases.

A recent study using SINAN data on self-harm in Brazil (2011-2022) reported an 822% nationwide rate increase (7.6 to 70.1/100,000), with higher rates among females but steeper annual increases among males ^10^. In that study, racial disparities shifted over time: Whites initially had higher rates than Black and Mixed-raced individuals, but by 2022, Mixed-race individuals had surpassed both, with Black/Mixed-race populations showing sharper annual rises. Regionally, rates peaked in 2019, dipped in 2020, and then rebounded, with the South and Central-West having the highest rates by 2022 ^10^. Our findings largely align with Alves et al. ^10^, but key differences emerge: the population aged 18 to 30 years in our data had higher rates than the general population nationwide, and White individuals in this subgroup had higher rates than Black/Mixed-race individuals, contrasting with the national pattern in which Mixed-race individuals led. Regional racial disparities were not analyzed by Alves et al. ^10^.

Sex differences in reported intentional self-inflicted injury in Brazil mirror global patterns: women exhibit higher rates of self-harmful behaviors, while men show higher mortality. This is attributed to men’s use of more lethal means, which increases the likelihood of achieving the intention to end their life ^10^ ^,^ ^37^. Moreover, women’s greater healthcare engagement and use of less lethal means reduce the likelihood of fatal outcomes ^10^ ^,^ ^38^. 

Our intersectional analysis revealed that White females maintained the highest national rates of intentional self-inflicted injury, followed by Mixed-race females, while Black males had the lowest. As noted, women have greater healthcare engagement, and in Brazil, White women are the ones who most seek healthcare services, followed by Black/Mixed-race women, whereas Black/Mixed-race males are the ones who seek the least ^38^. These differences in healthcare engagement may explain the rate differences, as seeking healthcare is necessary for an intentional self-injury case to be reported. Regionally, patterns varied considerably. 

In the North, Northeast, and Central-West, Mixed-race females consistently had the highest or second-highest rates by the end of the study period, with White females typically ranking second. These regions also showed the most dramatic rises among Mixed-race populations, especially in the Northeast and Central-West. The increasing rates in these regions may be linked to precarious living conditions and limited health services − especially with a lower coverage of public mental health services compared to the South and Southeast − among other aspects that influence well-being ^39^ ^,^ ^40^ ^,^ ^41^. 

In contrast, the Southeast and South exhibited distinct trends: White females ultimately led in both regions, though the Southeast initially had Black females as the group with the highest rate in 2014, and the South was notable for its steep increase among Black males. Notably, Mixed-race females in the Southeast recorded the greatest rate increase in this region. These regional differences suggest that demographic, socioeconomic, and cultural factors differentially influence vulnerability across Brazil’s diverse populations. For example, in the Southeast, an urbanized and industrialized region, the initial predominance of Black women (2014) may reflect the overlap of structural racism and gender vulnerabilities in peripheral areas, while the subsequent rise among White women (in 2023) may reflect greater visibility and diagnosis of mental health issues among populations with better access to health services, which have historically excluded Black and Mixed-race populations ^38^ ^,^ ^42^.

Compared with White males, Mixed-race and White females emerged as the most vulnerable groups for intentional self-inflicted injury among individuals aged 18-30 years, nationwide and in most regions, along with Black females in the South. White and Black/Mixed-race females also show higher healthcare access rates than their male counterparts ^38^, which may contribute to these disparities, given that reporting depends on seeking healthcare services.

This study has several limitations that should be considered when interpreting the results. Intentional self-inflicted injuries are likely underreported in Brazil despite mandatory reporting, potentially leading to underestimated rates. Moreover, the SINAN dataset presents several limitations. The lack of data on the type of intentional self-inflicted injury (suicidal or non-suicidal), due to missing data or poor completion of the reporting form, makes it impossible to properly classify cases as suicide attempts or non-suicidal self-inflicted injury. Even though these outcomes share similar risk factors ^13^, it is important to distinguish whether the intentional self-inflicted injury was suicidal or not, to provide a better understanding of the etiology of each outcome. Moreover, while socioeconomic inequalities are linked to self-injurious behaviors (suicidal or not) ^11^ ^,^ ^12^ ^,^ ^13^, our analysis could not assess these factors due to limited data on educational attainment and concerns about bias from missing data.

Despite these limitations, our study is one of the few that estimates rates using SINAN data on intentional self-inflicted injury. Most studies using this dataset only describe sample characteristics and compare groups ^6^ ^,^ ^8^, without population estimates, which limits comparability beyond the sample. Alves et al. ^10^ did provide population estimates for self-harm notifications for the Brazilian population aged 10 years or older; however, no rates were estimated for the intersection of race/skin color and sex. The lack of studies with comparable measures, especially those employing an intersectional approach, limits the discussion of our findings. 

## Conclusion

To the best of our knowledge, this is the first study to assess and compare intentional self-inflicted injury rates and trends among Brazilians aged 18-30 years nationwide and across all regions, using an intersectional approach considering race/skin color and sex over a ten-year period (2014-2023), utilizing a national database. 

This study reveals a concerning escalation in intentional self-inflicted injury rates among this population over the past decade, particularly among Mixed-race and White women. The intersectional analysis demonstrated that sex and race/skin color interact to produce health disparities, with marked regional differences. These findings underscore the urgent need for self-injurious behaviors prevention policies that are both universal and selective ^43^ ^,^ ^44^.

Universal policies should focus on expanding the coverage of mental health services, including Centers for Psychosocial Care (CAPS) and primary care services, which represent the first line of access to mental health care and professionals in the SUS. Selective policies holding intersectional and territorial sensitivity should focus on strategies such as targeted awareness campaigns, professional training, and strengthening psychosocial care, prioritizing the most affected groups − especially Black and Mixed-race women in regions with the steepest rate increases. 

Moreover, it is recommended that the Brazilian Ministry of Health, along with state and municipal health departments nationwide, develop strategies to standardize the completion of the SINAN form and reduce missing data for variables such as educational attainment, gender identity, and type of injury (whether suicidal or not). 

Given the lack of studies using comparable measures in the same database, further research is necessary. Future studies should incorporate an intersectional approach along with socioeconomic and qualitative variables to deepen understanding of the underlying factors driving these trends.
