# Supplementary material for: Intersection of race/skin color and sex in intentional self-inflicted injury rates among Brazilian people (2014-2023)
Source: Cad Saude Publica. 2026 Jan 1;42:e00097725. doi: 10.1590/0102-311XEN097725 (PMC12928197; doi:10.1590/0102-311XEN097725)
Supplement: Supplementary Material [file 1678-4464-csp-42-EN097725-s.pdf]

Supplementary Material

Table S1 Dataset characteristics of reported intentional self-inflicted injury from 2014 to 2023 among adults (18-30 years) in Brazil.

Variables	Year									
	**2014 [n = 10,710]**	**2015 [n = 14,002]**	**2016 [n = 15,873]**	**2017 [n = 24,016]**	**2018 [n = 33,081]**	**2019 [n = 49,037]**	**2020 [n = 39,506]**	**2021 [n = 47,068]**	**2022 [n = 55,466]**	**2023 [n = 75,005]**
	**%**	**%**	**%**	**%**	**%**	**%**	**%**	**%**	**%**	**%**
Sex										
Male	34.8	36.2	35.3	34.2	34.0	31.8	33.5	32.5	32.6	32.7
Female	65.2	63.8	64.7	65.8	66.0	68.2	66.5	67.5	67.4	67.3
Missing	-	-	-	0.02	0.01	0.02	0.04	0.01	0.02	0.04
Race/skin color										
White	48.0	45.5	46.7	48.1	47.9	45.9	44.5	41.9	41.4	41.6
Black	6.7	6.7	6.2	6.4	6.0	6.1	6.6	6.6	6.8	7.5
Brown	30.5	31.9	32.8	34.5	35.5	37.3	37.9	40.5	42.4	44.1
Other*	1.5	1.4	1.3	1.4	1.3	1.4	1.6	1.7	1.7	1.6
Missing	13.3	14.5	13.0	9.7	9.2	9.3	9.3	9.3	7.8	5.2
Educational attainment (in years)										
< 8	18.4	17.4	15.0	13.4	11.9	10.5	10.6	9.4	8.0	8.2
≥ 8	42.5	42.1	43.5	48.6	52.0	52.5	50.7	50.3	51.7	54.6
Missing	39.2	40.4	41.6	38.0	36.2	37.1	38.6	40.3	40.2	37.3
Race/Skin color and sex intersection										
White male	16.7	16.7	16.1	16.0	15.9	14.3	14.4	13.1	13.0	13.2
Black male	2.3	2.5	2.4	2.4	2.3	2.1	2.4	2.3	2.4	2.7
Brown male	10.7	11.6	11.9	12.2	12.3	11.9	12.7	13.4	14.0	14.6
White female	31.3	28.8	30.6	32.1	32.0	31.6	30.1	28.9	28.4	28.4
Black female	4.3	4.2	3.8	4.0	3.8	4.0	4.2	4.2	4.4	4.8
Brown female	19.8	20.3	20.9	22.2	23.2	25.4	25.2	27.1	28.5	29.6
Missing	14.8	15.9	14.3	11.1	10.6	10.7	11.0	11.0	9.4	6.9

* Other: Indigenous or yellow.
